# Life threatening abscess in the visceral space with penicillin and metronidazole resistant Prevotella Denticola following use of a laryngeal mask airway: case report

**DOI:** 10.1186/s12871-021-01322-9

**Published:** 2021-04-05

**Authors:** Hervé Vanden Eede, Elizabeth Norris, Michaël Torfs, Olivier Vanderveken

**Affiliations:** 1 Department of anaesthesiology and intensive care, AZ Rivierenland, Rumst, Belgium; 2 General practitioner and consult in medical education, Bath, UK; 3 Department of radiology, AZ Rivierenland, ‘s Herenbaan 172, 2840 Rumst, Belgium; 4 Head of the department Nose Throat Ear surgery, University hospital Antwerp, Drie Eikenstraat 655, 2650 Edegem, Belgium

**Keywords:** Laryngeal mask airway, Abscess, Visceral space, Prevotella denticola, Case report

## Abstract

**Background:**

Laryngeal mask airways (LMA) are commonly used for airway management. Complications with this device are rare. However, when they do occur, there is a high risk for respiratory problems, necessitating early diagnosis and treatment. We present the first case of a life-threatening abscess spreading in the visceral space caused by a penicillin and metronidazole resistant Prevotella Denticola after the use of an LMA.

**Case presentation:**

A female patient was admitted to our day care centre for bunion surgery. A single use LMA size 3 (Solus®, intersurgical, Wokingham, Berkshire, United Kingdom) was successfully inserted. After surgery, the patient complained of a sore throat and amoxicillin was prescribed by the general practitioner. Three days after surgery the patient was admitted to the Intensive Care Unit (ICU) for obstructive breathing, due to an abscess in the visceral space. Retropharyngeal and certainly parapharyngeal abscesses in adults are already rare. This case however, is unique because it is the first case of abscess spreading into the visceral space after the use of an LMA. Amoxicillin/clavulanate and vancomycin were started. The abscess was incised 5 days later and microbiology showed 3 positive cultures of the anaerobe Prevotella denticola, resistant for penicillin and metronidazole, but sensitive for amoxicillin/clavulanate. The patient fully recovered.

**Conclusion:**

LMA’s are easy to use and are established, safe tools to support ventilation of the airway. In this case, the authors hypothesise a small wound in the lateral pharyngeal wall probably created an opening into the visceral space causing infection with Prevotella denticola, supporting the idea that the pharyngeal mucosal space must be part of the visceral space. Additionally, early recognition and treatment of an LMA induced abscess is necessary to prevent evolution of complications leading to airway obstruction.

## Background

The laryngeal mask airway was developed by Doctor Archie Brain in 1980 as a safe alternative to endotracheal intubation and was introduced into clinical practice in 1988 in the United States [1]. This device can be placed without a laryngoscope or muscle relaxation and can be used for both spontaneous breathing and controlled mechanical ventilation. The mask was designed to adapt to the contours of the hypopharynx with its lumen facing the laryngeal opening. The distal tip of the LMA cuff presses against the upper oesophageal sphincter, its sides face into the pyriform fossae and the upper border rests against the base of the tongue. The formation of a low-pressure seal around the glottis provides a safe and effective artificial airway. Different comparison studies between the laryngeal mask airway and tracheal intubation were performed [2, 3]. The LMA became adopted into worldwide practice because of the easy and successful insertion (99,81%) and the low rate of critical incidents (0,15%) [4]. The 4TH national audit project (NAP4) in 2011 estimated that 56% of general anesthetics performed were carried out using supraglottic airway devices (SGAs). Anaesthesia events led to 16 deaths and three episodes of persistent brain damage: a mortality rate of 5.6 per million general anaesthetics, one per 180,000. Rates of death and brain damage for different airway devices (facemask, supraglottic airway, tracheal tube) varied little [5].

Indications and contra-indications for the use of laryngeal mask airway [6] are listed in Table 1.

**Table 1:** Indications and contraindications of LMA

Indications	primary airway management device in the operative setting in pre-selected, fasted patients a temporary bridge to intubation by pre-hospital providers in cardiac arrest situations a rescue device in “can’t intubate, can’t oxygenate” alternative to the use of bag valve masks to reduce the risk of gastric inflation
Contraindications	a conscious or awake patient poor pulmonary compliance high airway resistance pharyngeal pathology a risk for aspiration and/or airway obstruction below the larynx.

The most commonly reported complications include: sore throat, laryngospasm, coughing, gagging, retching, vomiting, glottis closure, arytenoid dislocation and vocal cord paralysis. There is still discussion about the advantages of the LMA for reducing respiratory complications compared to the endotracheal tube. A systematic review performed by Yu in 2010 showed that, for the patients receiving general anaesthesia, the use of the LMA resulted in a statistically and clinically significant lower incidence of laryngospasm during emergence, postoperative hoarseness and coughing in comparison to an endotracheal tube (ETT) [7]. However, a recent systematic review showed no clear advantage of the LMA over ETT in incidence of postoperative airway complications. Only the LMA Supreme® (Teleflex, Westmeath, Ireland) may reduce this risk [8]. This indicates that this could also be the best choice of LMA. However, there are controversial reports about the benefits of one specific LMA over others regarding the incidence and severity of pharyngolaryngeal complications and more studies need to be done to prove this hypothesis. The i-gel (Intersurgical), invented by Chang is reported to cause less oropharyngeal mucosal injury than the LMA Protector. It is an anatomically preshaped LMA with a soft, gel-like noninflating cuff. However, the incidence of postoperative sore throat in this comparative study between the i-gel and the LMA Protector remained the same. The oropharyngeal mucosal injury in this study was evaluated by checking for presence of blood on the LMA’s surface after its removal [9]. Furthermore, in another study, one day postoperatively after general anesthesia lasting less than two hours, the i-gel, the LMA Unique and LMA Supreme were reported to cause a similar incidence of sore throat [10].

Severe respiratory complications due to retropharyngeal abscesses following injuries caused by LMA are rare [11–16]. The extension of the abscess into the parapharyngeal space is even more rare. Only two of these cases have been reported [17, 18]. We report the first patient with an abscess in the visceral space following the use of an LMA. This case is unique because of the localisation of the infection, supporting a hypothesis that the pharyngeal mucosal space must be part of the visceral space [19–21]. Secondly, the infection became life-threatening despite timely treatment with antibiotics due to an infection with a penicillin and metronidazole resistant Prevotella denticola.

## Case presentation

### Patient information

A 61-year-old female patient with a body weight of 67 kg was admitted in our day care centre for bunion surgery. Her medical history included hypertension, hypercholesterolemia, a surgical history of appendicectomy and she smoked 5 cigarettes a day. She was compliant with her medication intake (amlodipine, simvastatin, prazosin, acetylsalicylic acid) and in a healthy condition. The patient had fixed dentures. Anaesthetic care was provided by an anaesthetist with a broad clinical experience of over 30 years. Anaesthesia was induced with 120 mg propolipid, 20 mcg sufentanyl and maintained with sevoflurane 2% in an oxygen-air mixture via spontaneous ventilation. A single use LMA size 3 (Solus®, intersurgical, Wokingham, Berkshire, United Kingdom) was successfully inserted after one attempt and the cuff was inflated with 20 ml of air. There was no documentation of difficult insertion, no complications during surgery and the duration of the procedure was 80 min.

### Clinical findings

The LMA was removed on recovery from general anaesthesia with the cuff deflated with evidence of a little blood on the LMA cuff. At discharge from day care centre the patient complained of a sore throat and a small amount of bloodstained sputum. This was not investigated any further and she was discharged with simple analgesics. The following day the pain in her throat became progressively worse and she presented to her general practitioner with fever, dysphagia and throat pain. Amoxicillin (500 mg (mg) 4x/day) and analgesics were prescribed as treatment for laryngitis. After three days symptoms persisted and the patient started to experience respiratory difficulty and worsening pain. The skin from the sternum bilaterally to both ears was swollen, red and warm and she was admitted to the emergency department.

### Diagnostic assessment

A contrast-enhanced computer tomography (CECT) scan of the neck and upper chest showed a large collection in the visceral space, with a mass effect leading to a deviation of the trachea to the left.

### Therapeutic interventions

The patient was referred to the ICU with dyspnoea. Oxygen therapy was started and the patient was initially treated with amoxicillin/clavulanate (1000 mg 4x/day) and vancomycin (1000 mg 4x/day) followed by surgical drainage under general anaesthesia 5 days later. After thorough examination by an otolaryngologist during surgery, a minor wound was found on the lateral pharyngeal wall. Microbiology showed 3 positive cultures of the anaerobe Prevotella denticola, resistant for penicillin and metronidazole, but sensitive for amoxicillin/clavulanate. After surgery, the patient had an uneventful recovery and was discharged a few days later. The patient fully recovered following discharge. The time-line regarding the evolution of this case is found in Table 2.

**Table 2:** Timeline: historical information about the evolution of this case

Time	Symptoms	Diagnosis	Treatment
Day 1	Sore throat, limited bloodstained sputum		
Day 2	Fever, dysphagia, throat pain	Clinical: laryngitis	Amoxicillin and analgetics
Day 5	Respiratory problems, worsening pain Red, warm, swollen skin from sternum to both ears	CT: abscess in the visceral space	ICU admittance Amoxicillin/clavunalate Vancomycine Fiberoptic intubation failed Tracheotomy not possible
Day 10	No more respiratory problems Diminished swelling	3 positive cultures: anaerobe Prevotella Denticola resistent for penicillin and metronidazole	Surgical drainage Wound on lateral pharyngeal wall
Day 14	Full recovery		Discharged from hospital

## Discussion and conclusion

This is a case of an abscess in the visceral space following LMA insertion for elective surgery, evolving into respiratory distress despite antibiotic therapy, with the need for admission to the ICU and incision and drainage of the abscess.

### The wound

Necrotic mucosal tissue and defects in the posterior wall after use of LMA have already been reported [22]. Soft tissue necrosis could have different causes such as: over-inflation, bad positioning, incorrect sizing of the LMA, prolonged direct pressure effects due to long procedural duration and direct trauma [23]. Direct trauma had even been reported to cause bleeding, uvular damage [24], tearing of the lingual frenulum [25] and ulceration of the soft palate [26]. Even with apparent easy insertion of an LMA such complications can occur. However they may be more likely if repeated attempts at insertion are needed or excessive force is used. Table 3 shows the possible sites, types and mechanisms of traumatic injuries caused by LMA [27].

**Table 3:** Sites, types and mechanisms of traumatic injuries caused by LMA (modified from Michalek)

Site of injury	Types of injury	Mechanisms of injury
Lips	Nerve injury Laceration	Compression by device, taping to device Direct trauma
Teeth	Displacement Fracture of roots	Direct trauma Biting on SGA/bite block
Tongue	Frenular injury Lingual nerve injury	Forceful or incorrect insertion Compression of lateral or inferior surface of the tongue by LMA
Uvula	Ischemia and necrosis	Direct trauma Prolonged compression
Epiglottis	Laceration Bruising	Anatomical abnormalities Forceful or oncorrect insertion
Pharyngeal mucosa	Laceration Bruising	Forceful insertion Inadequate lubrification Prolonged insertion Too high cuff pressures
Laryngeal apparatus	Arytenoid dislocation Recurrent laryngeal nerve injury	Direct trauma Compression of the nerve in the piriform fossa

Since the patient complained of sore throat with bloodstained sputum on the day of surgery, we believe that blunt trauma to the lateral pharyngeal wall was caused by the tip of the laryngeal mask during insertion. This pharyngeal tear created a entrance for the Prevotella into the visceral space, leading to abscess formation.

Another cause in our case could have been over-inflation or bad positioning. Unfortunately, cuff pressures are not routinely measured in our institution. Therefore, relative over-inflation cannot be excluded as a cause of pharyngeal mucosal injury. However, the limit of 20 ml (milliliter) for the cuff for a size 3 as suggested by the product instruction leaflet was not exceeded. Nevertheless, routine manometry for LMA should be the standard of care [28]. We do not believe that the injury was caused by a larger than necessary LMA [29] because a size 3 was used for a female of 67 kg. Bad positioning is difficult to exclude, but there was no recorded ventilatory problem during anesthesia and there was no notification of bad positioning at removal of the LMA. However, it has already been described that 40–60% of LMA are not correctly positioned [30]. It is our belief that using a manometer should be mandatory to avoid excessive pressure, certainly in operations with a longer duration (> 1 h). Seet et al. showed that the incidence of postoperative pharyngolaryngeal adverse events could reduce by 70% while using intraoperative manometry to measure and adjust the intracuff pressure to less than 44 mmHg (millimeters of mercury) or 60 cm H_2_O (centimeters of water) in spontaneously breathing patients [31]. However, with the knowledge that the correct position of the LMA is only achieved 50–60% of the time [30] and that various reasons for trauma have been pointed out [27], the outcome of the patient would probably not have changed.

### The infection

The patient was otherwise healthy and immunocompetent.

Retropharyngeal abscesses in adults are rare. Tennebaum reported 51 cases in 1996 in his review. Forty one percent of these cases involved patients with a recent history of pharyngeal tear by a preceding procedure or impacted foreign body [32]. Harkani reported 5 case reports in a review in 2011 which occurred mostly due to local trauma due to foreign body ingestion or in immunocompromised patients [33].

Parapharyngeal abscesses in adults are even more uncommon. Sethi found only 9 patients in his review [34]. All the patients in this study presented with fever and swelling of the upper neck and all needed exploration, incision and drainage. Infection in the parapharyngeal space can spread to the retropharyngeal space and the mediastinum [35], requiring urgent diagnosis and treatment. Immunocompromised patients and diabetics are also more prone for this kind of infections [36]. However, our patient had an abscess in the visceral space. How could this deep abscess originate from the insertion of a LMA? As discussed earlier, the otolaryngologist found a small wound in the lateral wall on the right side. The spread to the visceral space can be explained by the anatomical boundaries of the visceral space. The visceral space has a controversial terminology with the main difference being the superior limit. Some authors consider the entire pharyngeal mucosal space, including nasopharynx and oropharynx, as a subcomponent of the visceral space [19–21] extending the superior border to the skull base, while others restrict the visceral space to the infra-hyoid neck, with the superior border at the level of the hyoid bone [37]. Our case report would support the first hypothesis. The pharyngeal mucosal space must be part of the visceral space, otherwise this type of abscess could not have occurred. Apart from this discussion, there is consensus that the visceral space extends to the superior mediastinum: this consensus is supported by our case report, as the superior mediastinum represented the inferior border of the abscess.

The strength of this case presentation is the combination of the chronology of events, the diagnosis on CECT of the visceral abscess and the positive microbiology. Limitations are the paucity of data to be found in the literature about the anatomical extension of the visceral space.

### The diagnosis

In our case the patient had a sore throat postoperatively. However, this is not uncommon after airway manipulation. A sore throat occurs in 12% after airway manipulation with 45% due to endotracheal intubation and only 18% attributed to LMA [38]. The cuff pressure has also an effect on the degree of pain [39]. Therefore, measurement of cuff pressure should be the standard of care in LMA use.

The evidence of some blood on the LMA at the time of removal could have alerted medical staff to the possibility of trauma caused by the LMA. Evidence exists that patients with a postoperative sore throat and bloodstained sputum should undergo more thorough clinical investigations [11]. In this patient, because only a small amount of bloodstained sputum was observed, no further investigation was initiated.

The patient presented to her general practitioner the following day with fever, dysphagia and throat pain. A common diagnosis of laryngitis was made and she was treated accordingly. After three days, the fever and pain persisted and the skin was swollen, red and warm from the sternum bilaterally to both ears. The patient was admitted to the emergency room where an abscess in the visceral space was confirmed after CECT scan before she was transferred to the ICU.

**Fig. 1:**
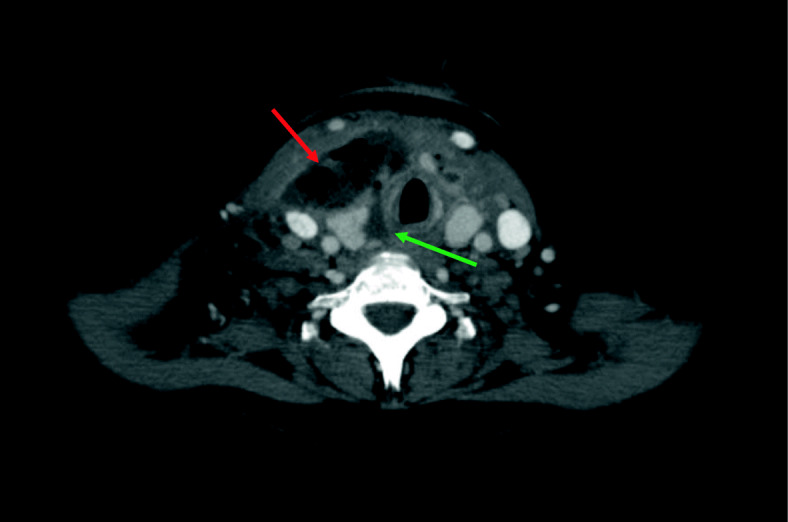
CECT, axial image. Large collection of fluid and air bubbles (hence, deep neck abscess) in the visceral space (red arrow). Draped over the thyroid gland, with a component between the right thyroid gland lobe and the trachea (green arrow), peripherally contained by the sternocleidomastoid muscle

The CECT scan of the neck and upper chest was performed 5 days after insertion of the LMA. The initial CECT scan shows a large collection of fluid and air bubbles (which proved to be an abscess) in a rather unusual location, called the visceral space. The collection pushes the right thyroid lobe and the right internal jugular vein posteriorly. The collection is therefore draped over the thyroid gland and contained by the overlying sternocleidomastoid muscle (Fig. 1, red arrow). Part of the abscess is located adjacent to the trachea on the right side, deep from the right thyroid gland lobe (Fig. 1, green arrow).

**Fig. 2:**
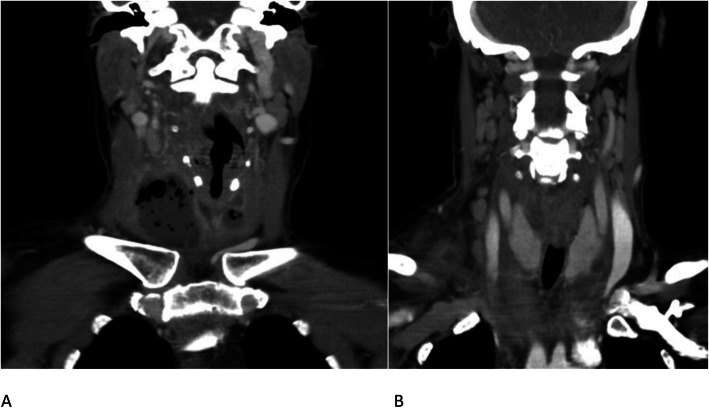
CECT, coronal image. Inferior extension of the deep neck abscess (largest part on the right side) into the border of the upper mediastinum with mass effect, leading to a leftward deviation of the trachea (**a**). Bilateral reactively enlarged jugulodigastric lymph nodes (**b**) without signs of intranodal abscess

There is inferior extension of the abscess into the border of the upper mediastinum, posterior from the manubrium of the sternum. The platysma is slightly thickened at the right side. Several bilateral jugulodigastric lymph nodes are reactively enlarged. Although some inflammation around the right external jugular vein is noted, this does not result in a frank thrombophlebitis. Tracheal deviation to the left can be seen on the coronal reconstructions due to mass effect of the abscess (Fig. 2).

Control CECT scan (not shown), obtained five days after the initial CECT scan, showed a decrease in diameters of the deep neck abscess.

### The treatment

In the day care centre only analgesics were prescribed. The general practitioner added amoxicillin 24 h later following national guidelines for the treatment of laryngitis. Unfortunately, the Prevotella spp. which caused the infection was resistant to amoxicillin. This made it possible for the infection to spread further causing pain, dysphagia and respiratory problems. Fortunately, the patient recovered well on therapy with amoxicillin/clavulanate and vancomycin. Respiratory distress improved over the following 24 h and the swelling diminished. Subsequently, the abscess was incised 5 days later under general anaesthesia. The treatment given, of antibiotics and surgical treatment for deep neck abscess was in accordance with international guidelines [40].

Prevotella is a genus of Gram-negative bacteria. They are non-motile, singular cells that thrive in anaerobic growth conditions. They thrive on tissues with decreased oxidation-reduction potentials, such as those with limited blood supply or tissue necrosis. Prevotella spp. are found in the oral cavity, the gastrointestinal tract and the vagina. They can act as an opportunistic pathogen, often penetrating tissues and establishing an infection at mucosal surfaces causing periodontal and tooth problems such as gingivitis and periodontitis. They predominate in periodontal disease and periodontal abscesses. Some Prevotella spp. are resistant to Beta-lactam antibiotics [41]. Effective antibiotic treatments are metronidazole, amoxicillin/clavulanate, ureidopenicilins, carbapenems, cephalosporins, clindamycin and chloramphenicol. This patient had extensive spread of the infection due to a late diagnosis of an abscess in the visceral space with a penicillin resistant Prevotella treated with amoxicillin.

#### Patient perspective on the received treatment

“I recovered from anesthesia of the first operation and was very pleased with the results of the operation. However, I had a sore throat when leaving the day care centre. One day later I was in so much pain that I had to call the general practitioner. I was thankful for the painkillers and I was well informed why antibiotics were prescribed. 24 hours later the pain and swelling was getting worse and I started to panic because I thought I would die. The treatment with oxygen and antibiotics started on intensive care and I was overwhelmed by all the actions the caregivers had to do to stabilise my situation. I am so glad I didn’t have to get a tracheotomy. Luckily, I have fully recovered and I am very thankful for the good treatment I have been given.”

## Conclusion

Laryngeal mask airways are easy to use and are established safe tools to support the airway. This is the first case report with a penicillin and metronidazole resistant Prevotella denticola as the documented bacterial cause of a deep neck abscess after LMA and the first case report of such an abscess in the visceral space.

In this case a small wound in the lateral pharyngeal wall potentially created an access from the pharyngeal cavity into the otherwise sterile visceral space causing infection with Prevotella denticola. The infection spread rapidly because of resistance to penicillin and metronidazole and caused respiratory problems necessitating admittance to ICU for respiratory failure. Early recognition and treatment of LMA induced abscess is necessary to prevent evolution to airway obstruction. If the patient complains of a sore throat and bloodstained sputum after evidence of blood on removal of the LMA, antibiotics should be considered and close monitoring of the patient’s ongoing condition should be mandatory.

Furthermore, we advise the routine use of a manometer when using LMA’s to obtain a cuff pressure of less then 44 mmHg to prevent soft tissue necrosis.

A randomised controlled trial to compare the incidence of infection and sore throat after findings of blood stains after LMA removal could be beneficial. Also because the LMA is usually inserted blindly, its exact position cannot be assured. Therefore, to place the device into the correct position, we need to understand the possible types of misplacement and have indirect detection methods of misplacement, and to master techniques to place the device correctly.

## Data Availability

The datasets used and/or analysed during the current study are available from the corresponding author on reasonable request.

## Abbreviations

LMA: Laryngeal mask airway
ICU: Intensive Care Unit
ETT: Endotracheal tube
Ml: Milliliter
Mg: Milligrams
MmHg: Milliliters of mercury
Cm H2O: Centimeters of water
CECT: Contrast-enhanced computed tomography scan
